# The promise and pitfalls of the internet for cognitive behavioral therapy

**DOI:** 10.1186/1741-7015-8-82

**Published:** 2010-12-07

**Authors:** Gerhard Andersson

**Affiliations:** 1 Linköping University, Department of Behavioural Sciences and Learning, Swedish Institute for Disability Research, Linköping, Sweden; 2 Karolinska Institutet, Department of Clinical Neuroscience, Center for Psychiatry Research, Stockholm, Sweden

## Abstract

Internet-administered cognitive behavior therapy is a promising new way to deliver psychological treatment. There are an increasing number of controlled trials in various fields such as anxiety disorders, mood disorders and health conditions such as headache and insomnia. Among the advantages for the field of cognitive behavior therapy is the dissemination of the treatment, being able to access treatment from a distance, and possibilities to tailor the interventions. To date, studies in which large effects have been obtained have included patient support from a clinician. Recent trials suggest that this support may come from non-clinicians and that therapist effects are minimal. Since studies also suggest that internet-delivered cognitive behavior therapy can be equally effective as face-to-face cognitive behavior therapy, this is a finding that may have implications for CBT practitioners. However, there are other aspects to consider for implementation, as while clinicians may hold positive attitudes towards internet-delivered CBT a recent study suggested that patients are more skeptical and may prefer face-to-face treatment. In the present work, I argue that internet-delivered CBT may help to increase adherence to treatment protocols, that training can be facilitated by means of internet support, and that research on internet interventions can lead to new insights regarding what happens in regular CBT. Moreover, I conclude that internet-delivered CBT works best when support is provided, leaving an important role for clinicians who can incorporate internet treatment in their services. However, I also warn against disseminating internet-delivered CBT to patients for whom it is not suitable, and that clinical skills may suffer if clinicians are trained and practice mainly using the internet.

## Introduction

Cognitive behavior therapy (CBT) is an established psychological treatment approach [1] that is regarded as evidence based for several health conditions such as anxiety disorders [2], major depression [3], and health problems such as insomnia [4], headache [5], and tinnitus [6]. Briefly, CBT is a treatment approach that encompasses assessment strategies, cognitive and behavioral treatment techniques, a collaboration between the patient and the clinician, and use of homework assignments [7]. In addition, there are numerous other techniques that are specific for conditions, such as exposure in the treatment of anxiety disorders [8]. While most CBT studies have used the individual treatment format there is plenty of evidence to suggest that CBT works well in the group format [9], and as guided self-help [10]. The most recent development in the field of guided self-help is the use of the internet to deliver this treatment [11], and this paper comments on the potentials and pitfalls of this development.

There has been a rapid expansion of outcome studies on internet-delivered CBT in the fields of psychiatry [12-14] and behavioral medicine [15], and there are an increasing number of studies showing that internet-delivered CBT can be equally effective as face-to-face CBT in conditions such as panic disorder [16], depressive symptoms [17], tinnitus [18], with studies on other conditions forthcoming.

This paper will highlight the impact the internet has had on CBT, its potentials and limitations, and projections into the future on how internet-delivered CBT will influence the dissemination and development of CBT. Understanding the pros and cons of internet-delivered CBT is important as it may increase access to CBT and hence reach more patients at a lower cost. However, while resources may be saved there is still a need for clinicians to guide the treatment if effects similar to face-to-face CBT are to be obtained.

## Discussion

While there are several studies on the effects of internet-delivered CBT, less is known about the realities of its implementation: that is, dissemination, acceptability and interest in internet treatments. Regarding dissemination there are several unguided open access programs available, but without the input from a clinician or other guiding person they tend to work less well than guided programs [12,14]. Open access programs without validated diagnostic procedures and guidance will probably not compete with face-to-face CBT in terms of efficacy, but can lead to increased interest in regular CBT after the internet treatment has been completed [19]. Unguided open access programs are also easily disseminated. Regarding dissemination in regular health care, there are now studies showing that internet-based treatment works under representative clinical conditions [20]. Acceptability and interest in internet-based CBT are constantly changing and may also differ between countries. Mohr *et al. *reported a study on 658 primary care patients in the US who were asked about their interest in different treatment formats [21]. Results showed that 91% would consider face-to-face treatment and only 48% internet treatment. Interests among clinicians may be greater, with one study showing that only a small minority felt that internet treatment is unacceptable as a treatment format [22].

### Impact on the field

Internet-delivered CBT has already and will continue to have a profound effect on CBT. The internet makes information about CBT more available [23], but the effective use of the internet to deliver CBT also raises questions for the CBT field. Training of therapists is regarded as important in CBT [24], but the evidence to date clearly suggests that therapist effects (in other words, does it matter who gives the support?) are minimal in internet-delivered CBT [25]. In a series of studies, Titov and colleagues have found that support administered from a technician can be equivalent to the support provided by a psychologist [26,27]. Indeed, this could be because in internet treatment the therapy is mainly presented via text and the computer program (which can include audio files, films, stories and so on), and hence the supportive role of the therapist in most cases will require less skills than in face-to-face therapies.

In CBT there has been a debate regarding the importance of tailoring treatment following a case conceptualization as opposed to using treatment manuals developed for specific diagnoses [28]. A recent development of internet-delivered CBT has been the use of tailoring, which makes internet treatment more suitable for comorbid conditions (such as the overlap between anxiety and depression), and also sensitive to patient preferences [29,30]. This is an example of how internet-delivered CBT may have an impact on regular manualized CBT, as there may be difficulties in moving between treatment manuals that are written for specific conditions in face-to-face therapies with patients who have comorbid conditions. One proposed solution has been to focus on the common transdiagnostic elements of CBT [31], and this has also been found to be effective when delivered via the internet [32]. The primary lesson for CBT could therefore be that computer support can increase therapist adherence to treatment protocols, increase the number of practitioners who can use CBT, and decrease therapist drift from manualized treatments [33]. Indeed a recent trial showed that that computerized support including web-based outcomes monitoring was a feasible approach to treat anxiety in primary care [34].

### Positive elements of increased access via the internet

There are several positive consequences of delivering CBT via the internet. The most obvious one is that it can be used to overcome distances. Many patients with anxiety and depression never get access to a trained CBT therapist, and the internet makes treatment more available for people who cannot travel to specialist clinics. Second, while internet treatment can be presented in real time [35], most treatment applications will not require that the work is performed at a particular time, and this is an advantage for both patients and clinicians. Clinicians may consult colleagues before responding to a question from a patient. Patients may work when it best suits them, and the therapy will not require the patients to take time from work to visit the therapist's office. Indeed, this resembles how internet banking has made it easier for many people who can access their bank affairs from home, and it is possible that patients in face-to-face CBT will gradually start asking for online support and material on the web when they are in treatment. However, internet treatment is still mainly a complement even if it can be a replacement. There will always be patients who will need face-to-face treatment or for whom internet treatment will complement other treatments such as pharmacotherapy or indeed face-to-face or group CBT. In addition, compliance with treatment may decrease when the treatment is provided from a distance, and in particular if very little support is given [14], which for some patients makes internet treatment less suitable.

### What has the spread of the internet added or removed from the field of CBT?

Since the treatment versus demand gap is large when it comes to most conditions for which CBT has been found to work [36], the development of internet-delivered CBT and the internet in general has probably helped to increase awareness of CBT to the general public, for example on the websites of mental health charities. Most likely internet treatment studies will gradually increase in number and there is now a tendency that novel treatment methods are tested in internet trials soon after that they have been developed [37], and that new treatment therapies will even be developed directly for use with the internet. These developments will also involve smart phones with special applications. While internet studies can be preceded by open smaller face-to-face or group trials [38], it will be more feasible to do the controlled investigations on the internet [37]. One additional aspect that the internet has added to the CBT field is the use of online questionnaires, and research has consistently shown that this administration format is feasible and works as well as paper and pencil questionnaires [39]. There are some potential risks with the dissemination of internet treatments. It could be argued that the clinical skills derived from actual practice with patients will deteriorate or even not develop if clinicians start doing most of their work via the internet. Another risk would be if internet treatments would dominate to the extent that regular clinical services would not be funded. As we have reasons to believe that face-to-face diagnostic interviews and examinations will be needed for many conditions (for example, diagnosis of major depression), the internet is not capable of replacing all aspects of good clinical management. A third risk is that internet treatments will be disseminated to the wrong patients who will experience treatment failure and then falsely conclude that CBT did not work for them. However, the opposing risk would be if internet treatments were not disseminated at all apart from a few specialist settings [20].

Finally, a projection into the future is that internet-delivered CBT will become integrated with other services in a clinician's toolbox. There may be room for large-scale open access programs, in particular intelligent systems which may be able to mimic what the online therapist is doing and thereby secure that effects are obtained [40]. Studies on guided self-help including internet studies may also reveal that previously less well known factors may be important for treatment adherence, such as the importance of having a deadline for completing treatment following which an interview is made [41].

## Conclusions

The internet has had a major impact for the field of CBT. First, information on the internet about CBT makes this treatment option better known. Second, the use of the internet for delivering CBT has been found to be effective in several randomized controlled trials [11]. Indeed, in line with the rapid spread of the internet at large, controlled trials on internet-delivered CBT have been conducted remarkably fast and it is telling that in some fields, such as treatment of anxiety disorders, that there are now more controlled studies on internet-delivered CBT than there are on the much older psychodynamic treatments for anxiety disorders [42]. Third, internet-delivered CBT raises questions of relevance for face-to-face CBT. Recent studies on internet-delivered CBT have found that the tailoring of treatment ingredients can be combined with a manualized approach. The role of the therapist providing support is less important in internet-delivered CBT and challenges the thinking regarding dissemination of CBT including training clinicians in CBT. Patient preferences for internet treatment will likely change in the very near future, although at present there are still a significant proportion who prefer the traditional face-to-face format [21].

Internet-delivered CBT may be used to increase adherence to treatment protocols and manuals. This will prevent 'therapist drift' away from structured treatments, and will also facilitate dissemination of CBT to other professionals than trained psychotherapists. In light of these observations, CBT professionals will probably benefit from using the internet in their clinical work, as one channel for communication and for improving the structure of information. Treatment standards will be needed in the future, as it can be hard for clinicians to discern what programs and approaches they should use. There is also a need to define standards for training clinicians using the internet in their work. Other potential challenges include the need to maintain clinical skills derived from face-to-face clinical practice, to secure reliable diagnostic procedures if clinical interviews are not feasible, and to find appropriate models for service delivery for internet-delivered CBT.

Practitioners and developers of internet-based CBT programs should consider the importance of proper patient diagnoses, evaluation of suitability and user friendliness of the internet system. Most treatments tested in research have been largely text based, but use of audio files and illustrative movies, web lectures and interactive features may help to make the treatment more accessible for persons who prefer other formats than pure text. As the internet and information technology change rapidly use of smart phones and their integration with the internet should now be considered when developing new systems and updating older programs.

## Competing interests

The author declares no competing interests. GA is a professor in clinical psychology and a licensed psychologist and CBT therapist http://www.gerhardandersson.se.

## Pre-publication history

The pre-publication history for this paper can be accessed here:

http://www.biomedcentral.com/1741-7015/8/82/prepub
